# PbAT: A user‐friendly R/Shiny platform for data‐driven decision support in crop improvement

**DOI:** 10.1002/aps3.70068

**Published:** 2026-06-29

**Authors:** Krishnan P. Abhijith, K. K. Vinod, Ranjith Kumar Ellur, K. T. Ravikiran, Rachit Kumar Saxena, Vignesh Muthusamy, S. Gopala Krishnan

**Affiliations:** ^1^ School of Crop Science, ICAR‐Indian Agricultural Research Institute Assam, Gogamukh Dhemaji 787035 Assam India; ^2^ Division of Genetics ICAR‐Indian Agricultural Research Institute 110012 New Delhi India; ^3^ ICAR‐Central Soil Salinity Research Institute Regional Research Station Lucknow 226002 Uttar Pradesh India; ^4^ Division of Crop Improvement ICAR‐Sugarcane Breeding Institute Coimbatore 641007 Tamil Nadu India

**Keywords:** experimental design, genotype × environment interaction, mating designs, multivariate analysis, trait analysis

## Abstract

**Premise:**

Modern plant breeding requires robust analysis of complex, multi‐environment datasets to identify superior genotypes. Although R offers powerful statistical packages, their use often requires programming expertise. Existing graphical user interface (GUI)‐based tools are either limited in scope or not openly accessible, leaving breeders without a comprehensive solution.

**Methods and Results:**

We introduce PbAT (Plant breeding Analytical Tools), an open‐access Shiny application that unifies multiple R packages into a seamless, code‐free workflow. PbAT enables trial design, data curation, experimental design analysis, stability testing, multivariate methods, and mating design analysis. Outputs include variance components, best linear unbiased estimators (BLUEs), best linear unbiased predictors (BLUPs), ANOVA tables, and publication‐ready plots, accompanied by automated plain‐language interpretations and model equations. Its modular architecture supports future integration of additional tools. PbAT is available online as a web application (https://pbat.online) and as an R package (https://github.com/abhijithkpgen/PbAT).

**Conclusions:**

By integrating powerful statistical methods with intuitive interfaces, PbAT streamlines plant breeding analytics, enhances reproducibility, and accelerates data‐driven decision‐making in crop improvement.

Plant breeding, being a continuous process, often generates extensive temporal and spatial datasets from field trials spanning across generations, seasons, and environments. Therefore, the success of a breeding program depends on critical decisions made from conclusions often drawn through robust statistical analysis of multi‐dimensional data (Cooper et al., 2025). Proper experimental design is critical not only for controlling experimental error and accounting for spatial field variability but also for ensuring that the non‐heritable noise does not obscure true genetic differences (Casler, 2015; Mathews and Crossa, 2022).

The analytical pipeline in modern plant breeding begins with the selection of a robust experimental design. This selection depends on the nature of the experimental design, field constraints, and the number of genotypes to be tested (Mathews and Crossa, 2022). Once the trial data are generated, the workflow requires rigorous data curation and outlier detection to ensure that biological signals are not obscured by technical errors or environmental noise. To accurately estimate genotype performance, contemporary approaches utilize linear mixed models, which effectively partition phenotypic variance into genetic and environmental components. This generates two primary types of estimates: best linear unbiased estimators (BLUEs), which are used when analytical scenarios treat genotypes as fixed effects to estimate specific group means, and best linear unbiased predictors (BLUPs), which are used when genotypes are treated as random effects to predict genetic merit and account for shrinkage (Yan, 2021; Holland and Piepho, 2024). In programs focused on hybrid development, this analytical framework extends to mating designs, which are indispensable for estimating combining abilities and identifying superior parental lines. Ultimately, robust decision‐making is achieved through stability and multivariate analyses, which allow breeders to characterize genotype‐by‐environment interactions (GEI) and untangle complex trait relationships before making final selection decisions (Olivoto et al., 2019).

R's extensive library of packages has made it a central tool for statistical research. Packages like lme4 (Bates et al., 2015) and sommer (Covarrubias‐Pazaran, 2016) are commonly used to fit linear mixed models and estimate fixed and random effects in experimental data. For multivariate analysis, FactoMineR (Le et al., 2008) and lavaan (Rosseel, 2012) provide efficient ways to generate both statistical summaries and plots. Additionally, ggplot2 (Wickham, 2011, 2016) offers the flexibility needed to produce high‐quality graphics within the R environment.

Plant breeding–specific R packages such as agricolae (de Mendiburu, 2023), augmentedRCBD (Aravind et al., 2020), and Stability (Yaseen and Eskridge, 2024) are commonly used for analyses of variance from the experimental data, including GEI studies as well as line × tester analyses, while DiallelAnalysisR (Yaseen et al., 2024) focuses on diallel methods. Metan (Olivoto and Lúcio, 2020) is widely applied for multi‐environment trial analysis and stability studies. Although several packages are available, such as agrostab (Cheshkova, 2019), correlation (Makowski et al., 2020), and GGEBiplots (Dumble et al., 2022), they function as standalone, unintegrated packages and are largely underutilized by users. Hussain et al. (2022) developed an R Markdown‐based pipeline that processes raw data and analyses with advanced statistical modeling and generates a comprehensive, interactive HTML report. However, to use these packages, the user needs to interact with R scripts.

A major limitation in utilizing R packages is the requirement for substantial statistical and computational expertise with the R ecosystem. To circumvent this barrier, graphical user interface (GUI) tools were introduced to provide a visual, code‐free layer that allows breeders to access statistical power through intuitive menus rather than complex scripting. GUI‐based plant breeding analytical software, such as Multi‐Environment Trial Analysis with R (META‐R; Alvarado et al., 2020), Analysis of Genetic Designs with R (AGD‐R; Rodríguez et al., 2015), Statistical Tool for Agricultural Research (STAR), and Plant Breeding Tools (PBTools; IRRI 2014), partially addressed these computational challenges. However, these applications suffer from specific limitations that hinder their wider adoption and long‐term utility. Tools such as STAR and PBTools face significant challenges regarding long‐term maintenance, as official support and updates have been discontinued. Furthermore, applications like META‐R and AGD‐R often lack extensibility and struggle with interoperability with other modern R workflows. Because they offer a restricted set of functionalities rather than fully integrated pipelines, breeders are frequently forced to switch between multiple software environments to complete standard multi‐environment and multi‐trait analyses. While robust statistical software like GenStat (Payne et al., 2009), ASReml (Butler et al., 2017), SPSS (IBM Corporation, 2020), and SAS (SAS Institute, 2011) offer broader functionalities for plant breeding data analysis, their accessibility is often impeded by significant licensing costs for many researchers and institutions in developing nations.

In developing Plant breeding Analytical Tools (PbAT), our goal is to systematically address these limitations. PbAT is a web‐based Shiny application (Chang et al., 2026) and is also available as a standalone R package. Our core objective with PbAT is to remove the barriers of advanced statistical analysis central to plant breeding by providing a freely available platform with a user‐friendly graphical interface. The application is structured into six main workflows covering trial design, trait exploration, analysis of experimental designs, stability analysis, multivariate analysis, and mating design analysis, covering a wide array of analytical procedures essential to breeding. These modules integrate features from several specialized R packages; therefore, the application provides plant breeders with a unified, open‐access platform.

In addition to running rigorous statistical computations, the PbAT application offers automated, plain‐language explanations of its graphs and results. This feature can help researchers connect the biological narrative of the data. In this paper, we describe the main modules and functionalities of PbAT and illustrate the utility of the application through a case study. Our objective is to demonstrate that PbAT can serve not only as an analytical tool, but also as a teaching and training platform that supports the next generation of plant breeders.

## METHODS AND RESULTS

### Overview

PbAT walks users through each stage of the breeding workflow with a straightforward interface, organized into six main analytical modules. The process begins with the Design Your Trial (DYT) module, which generates field layouts and field books from the technical details input by the user. Once the experiments are conducted and the data are generated, users can switch to the Trait Explorer (TEX) module for necessary exploratory data analysis and data quality checks. The curated data can then be imported to the Experimental Design Analysis (EXA) platform to analyze breeding designs such as randomized complete block design (RCBD), completely randomized design (CRD), and augmented RCBD and alpha‐lattice designs. PbAT incorporates both descriptive as well as mixed model analyses within this workflow.

Following the primary analysis, the generated outputs, such as BLUEs and BLUPs, can be utilized in the Multivariate Analysis (MVA) module to perform principal component analyses (PCA), correlation, and path analysis. The same outputs can also be fed to conduct stability analysis via the separate Stability Analysis (STA) module, where the software implements additive main effects and multiplicative interaction (AMMI) and genotype plus genotype‐by‐environment (GGE) biplots. Additionally, users can investigate the combining ability of parental lines through the Mating Design Analysis (MDA) module, which includes diallel (Griffing's methods 1–4; Griffing, 1956), partial diallel, and line × tester analyses. To provide an easy interface and connectivity between analyses, stability analysis and selected multivariate analyses can be performed either independently or directly integrated into the experimental design workflow, as the pipeline utilizes the output from the initial analysis as an input for subsequent analyses.

PbAT is also available as a standalone R package and allows users to run all analyses locally on their computer. The application generates the following outputs: summary tables, high‐quality plots, automated textual interpretations of the results, and CSV files. The application is developed in R 4.5.0 (R Core Team, 2025), incorporating the Shiny framework for GUI‐based deployment (Chang et al., 2026). The online version of this application can be accessed at https://pbat.online. The R package version of PbAT can be run natively on a user's computer and with all data and code for release v1.0.5 available at Zenodo (Abhijith et al., 2025; 10.5281/zenodo.17020132). It can also be installed directly in R by running the script:

*library(devtools)*

*devtools::install_github(“abhijithkpgen/PbAT”)*

*library(PbAT)*

*run_app()*

### Conceptualization and key uses

PbAT was developed to consolidate diverse analytical approaches in plant breeding into an integrated open‐access platform, harmonizing statistical workflows with the practical needs of breeding programs rather than serving merely as a computational tool. The application is organized around four core support systems: pre‐trial design support, data curation and analytical support, decision support, and pedagogical support.

Pre‐trial design support is incorporated in the first module, which helps formulate trials by implementing the standard experimental and mating designs widely employed in plant breeding. The second core component, post‐trial data curation and analyses, involves tools for spatial trait exploration, data quality checks, and outlier detection. This component generates essential statistical outputs, including analysis of variance (ANOVA) tables, variance components, and adjusted means (BLUEs and BLUPs) necessary for downstream evaluation. The third core component, the decision support system, guides the user to further explore phenotypic response patterns through multivariate methods and stability models, aiding the evaluation of genotype performance across environments. This has tremendous potential for application in plant breeding as multi‐environment trials are central to cultivar development programs across the globe. These trials identify stable and high‐yielding genotypes for varietal release and recommendations (Chauhan et al., 2016).

In addition to its research applications, PbAT also offers strong pedagogical value, the fourth core component of the application. The application's workflows not only display the underlying model equations but also generate plain‐language interpretations of results, allowing learners and trainees to experience the complete process of deriving end‐to‐end outcomes. To further support this, the help section is designed with clear textual instructions and sample datasets that can be readily applied across different modules. These features can be very convenient for training activities in plant breeding and quantitative genetics, especially for classroom instruction and capacity‐building programs at institutions with limited access to advanced statistical training or proprietary software.

### System architecture and analytical workflow

The core of the application architecture relies on Shiny modules, which unify both the user interface and the server‐side logic for each distinct workflow. This modular structure helps to segregate different functionalities, making the application easier to debug, test, and scale with new features without disrupting existing code or workflows. The Shiny reactive programming model supports the interactivity of the application, and this ensures user interface components are dynamically linked to the back‐end analytical functions. Therefore, any changes made with the user input, such as selecting a new analysis or uploading a different dataset, will automatically trigger the relevant computations. The outputs are updated in real time, ensuring a seamless user experience. To create a simple environment for workflow selection and data upload, the user interface has been developed with Shiny and customized cascading style sheets (CSS).

Integrated into the core support systems, PbAT users can access the six main workflow domains from the home panel, namely, Design Your Trial (DYT), Trait Explorer (TEX), Experimental Design Analysis (EXA), Stability Analysis (STA), Multivariate Analysis (MVA), and Mating Design Analysis (MDA) (Figure 1). The input data are typically uploaded in CSV format. Prior to the analysis, users must carefully inspect the data format, as the format required for each of the analytical modules may differ slightly based on the factors involved. The tutorial data provided under the Help and Guide tab can be taken as the reference for choosing the appropriate format and can be opened and edited in spreadsheet applications. Analysis starts with selecting an appropriate analysis workflow; after data upload, clicking the Proceed to Analysis button directs the user to the corresponding analysis tabs. The workflows incorporate several R packages such as lme4 (version 1.1‐37; Bates et al., 2015) and emmeans (version 1.11.2‐8; Lenth et al., 2026) for linear mixed models, metan (version 1.19.0; Olivoto and Lúcio, 2020) for stability analysis, FactoMineR (version 2.12; Le et al., 2008) and lavaan (version 0.6‐19; Rosseel, 2012) for multivariate procedures, as well as other supporting packages (Table S1 in Appendix S1).

**Figure 1:**
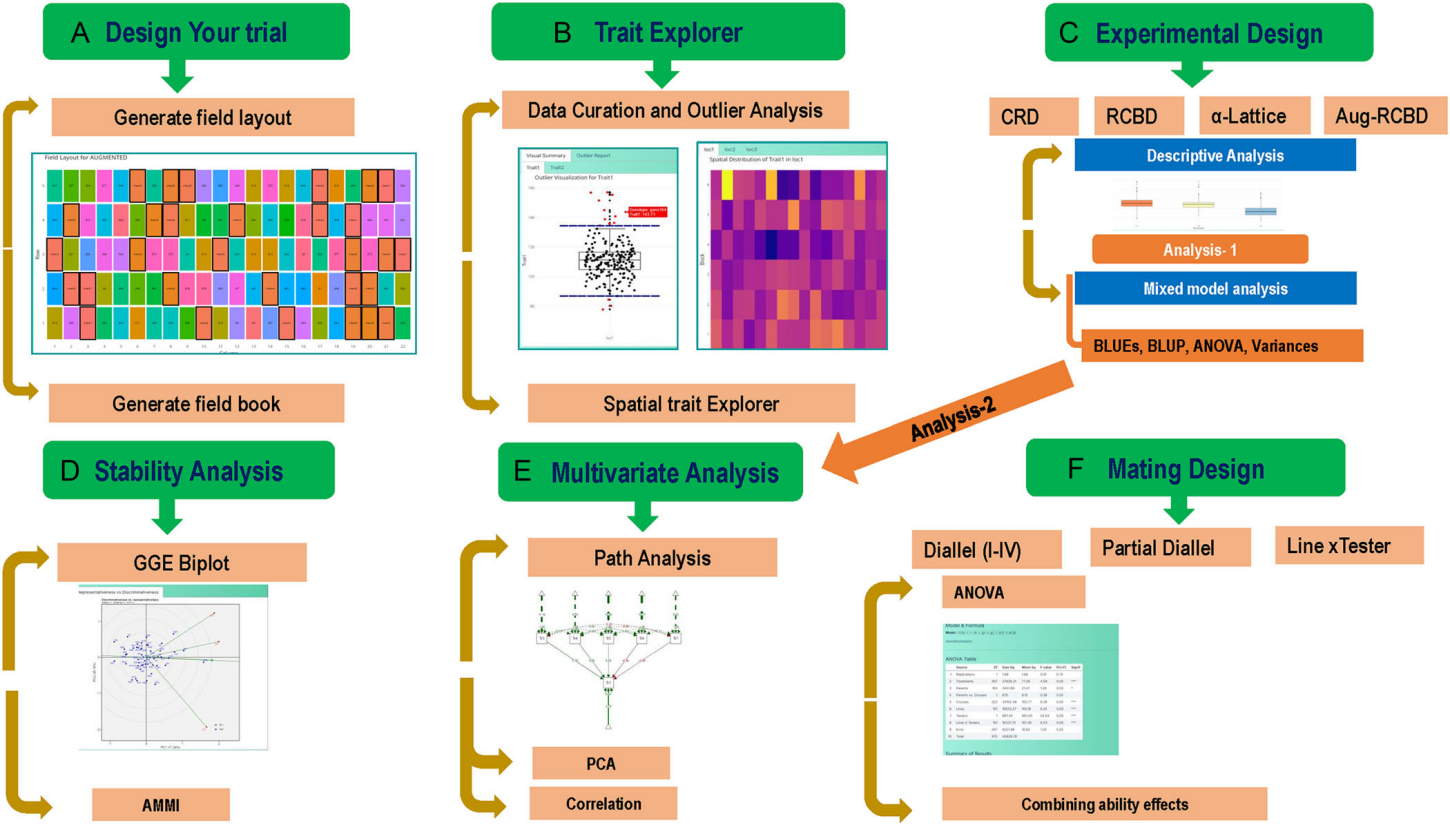
Schematic overview of the six core analytical workflows in PbAT. (A) Design Your Trial: Generates randomized field layouts (visualized) and field books for complex designs. (B) Trait Explorer: Facilitates data quality control through outlier detection boxplots and spatial distribution heatmaps. (C) Experimental Design Analysis: Executes descriptive and mixed‐model analyses (e.g., alpha lattice, RCBD) to derive BLUEs/BLUPs. (D) Stability Analysis: Utilizes the output from the experimental design module (indicated by the Analysis‐2 arrow) to generate GGE and AMMI biplots. (E) Multivariate Analysis: Performs path analysis, PCA, and correlation studies using adjusted means. (F) Mating Design Analysis: Estimates combining ability effects and ANOVA for diallel and line × tester populations.

On landing on the Analysis tab, the user is required to select details of various analytical factors from the pull‐down menu on the sidebar. The menu items are automatically populated from the column headers in the data file. The user can select appropriate models, methods, or analyses and proceed to run the analysis. Analysis outputs are generated online mainly in HTML format and are downloadable as TXT, CSV, and PDF files. Each of these analyses generates a set of outputs that may vary with the workflow chosen and can be downloaded as compressed archives in ZIP format. PbAT has been developed under R 4.5.0 and tested on R 4.3.0–4.5.0 (R Core Team, 2025). Earlier versions of R may not support certain dependencies, and therefore an updated environment is recommended for reliable performance.

#### Design Your Trial (DYT) workflow

The design of robust experimental trials is the foundation of any project, and a successful trial begins with a statistically sound experimental design. The principles of experimental designs, experimental units, replication, blocking, and randomization fundamentally shape the outcome of downstream statistical analysis and any resulting conclusions (Casler, 2015). The PbAT DYT workflow has been carefully developed to generate randomized and optimized layouts for the common designs used in plant breeding trials. Here, users can create field layouts through a simple, interactive process that guides them in deciding the number of replication/blocks based on the number of genotypes being tested, to ensure statistical robustness.

The workflow is relatively simple, and users can begin by uploading (or pasting) genotype names only or, in the case of augmented RCBD, genotype names along with checks in a CSV file as column(s); users then select the desired experimental design from a dropdown menu, with options including RCBD, alpha lattice, and augmented RCBD. Users also have an option to create a set of genotypes with custom prefixes and then generate a layout, which could be very handy in training programs for a quick demonstration. The application can automatically detect the number of genotypes (as well as the number of checks in the case of augmented RCBD). Based on this, the application guides users to decide the minimum number of blocks to be selected. Once the parameters are entered, the application generates a fully randomized field layout. The output is provided as a field layout in PDF format, and the field book containing the complete field plan is provided as a CSV file, with columns for plot number, replication, block, and assigned genotype. The layout file can be taken directly to the field for implementation during field preparation, and the field book can be used for data collection.

#### Trait Explorer (TEX) workflow

The TEX workflow serves as a critical checkpoint for data quality control and initial exploratory analysis. Although it is a preliminary analysis, this step is essential because it aids researchers in gaining a fundamental understanding of their dataset, identifying potential anomalies, checking for data entry errors, and assessing the overall distribution of traits. PbAT offers an interactive module to detect outliers within the TEX workflow. Here, users are given a choice to detect outliers within the environment or globally (in multi‐environment trials). Users can detect outliers using either a standard interquartile range (IQR) method (Najafabadi et al., 2023) or a standard deviation (SD) cutoff (Lehmann, 2013; Mahajan et al., 2020). While the IQR method applies a fixed standard threshold, the SD method is user‐configurable; the software provides a default recommended cutoff of 3.0, which users can manually adjust to suit their specific data distribution. The analyses produce interactive boxplots for each environment, with potential outliers clearly flagged. All the flagged data points are indicated in the report, and users can download the report and proceed with manual curation.

In the plant breeding trials, soil heterogeneity and microenvironmental conditions can create gradients or patches that can significantly influence crop performance (Liu et al., 2021). This spatial variation introduces non‐heritable noise that can obscure true genetic differences between genotypes and eventually leads to incorrect selection decisions (Cursi et al., 2021). The Spatial Trait Explorer (sTEX) module in this workflow provides an interactive heatmap of the experimental field, which could help breeders to visually identify patterns of spatial variability for the selected traits.

#### Experimental Design Analysis (EXA) workflow

The EXA workflow is one of the core statistical engines of PbAT. This workflow is developed to analyze data from both single‐ and multi‐environment trials. The fundamental purpose of this module is to accurately partition the observed variation into genetic, environmental, and residual components, thereby assessing genotype performance through precise estimates.

The EXA workflow integrates field experimental designs, RCBD, CRD, alpha lattice, and augmented RCBD. The analysis workflow follows a two‐step process: descriptive statistical analyses and model‐based analysis. The module computes and displays key descriptive statistics and generates visualizations such as boxplots and Q–Q plots to explore the variance distribution among the traits within the experimental population. This step makes use of the lme4 package to fit linear mixed models (Bates et al., 2015) and the emmeans package to estimate the adjusted means of genotypes (Lenth et al., 2026). PbAT allows users to specify various models (fixed or random) depending on the analytical scenario. When evaluating specific, non‐random sets of genotypes (such as standard checks or specifically selected lines), genotypes are used as fixed‐effect factors to generate BLUEs obtained as estimated marginal means using the emmeans package. Conversely, when the objective is to predict the genetic merit of a population sampled from a larger reference pool, genotypes are taken as random effects to generate BLUPs. All the model equations incorporated for model‐based analysis of the field trials are presented in Table 1. In both cases (fixed and random), the workflow also performs a likelihood ratio test (LRT) to evaluate the significance of random effects along with their *P*‐values. To simplify this complex environment of outputs, we have incorporated a dynamic interpretation block, which produces a plain‐language summary of the results. The interface is further divided into analysis 1 and 2 within this module. The adjusted means (BLUEs or BLUPs) generated from the EXA workflow can serve as the direct input for subsequent MVA and STA workflows.

**Table 1:** Model equations applied in PbAT for the analysis of single‐ and multi‐location plant breeding trials using randomized complete block design (RCBD), alpha lattice, and augmented experimental designs.

Design	Single‐environment trial model	Multi‐environmental trial model	Reference
RCBD	$\;Y_{\mathrm{ij}}=\;\mu +g_{i}+r_{j}+\varepsilon_{\mathrm{ij}}$	$Y_{\mathrm{ijk}}\;=\;\mu +g_{i}+e_{k}+{\left(\text{ge}\right)}_{\mathrm{ik}}+r_{j}(k)+\varepsilon_{\mathrm{ijk}}$	González‐Barrios et al. (2019); Ellur et al. (2025)
Alpha lattice	$Y_{\mathrm{ijk}}=\;\mu +g_{i}+r_{j}+b_{k}(j)+\varepsilon_{\mathrm{ijk}}$	$Y_{\mathrm{ijkl}}=\;\mu +g_{i}+e_{\;l}+{\left(\text{ge}\right)}_{\mathrm{il}}+r_{j}(l)+b_{k}(\text{jl})+\varepsilon_{\mathrm{ijkl}}$	Bourke et al. (2018); Nannuru et al. (2022)
Augmented design	$Y_{\mathrm{ij}}=\;\mu +g_{i}+r_{j}+\varepsilon_{\mathrm{ij}}$	$Y_{\mathrm{ijk}}=\;\mu +g_{i}+e_{k}+{\left(\text{ge}\right)}_{\mathrm{ik}}+r_{j}(k)+\varepsilon_{\mathrm{ijk}}$	Gutiérrez et al. (2015); Garin et al. (2020)

*Note*: Y_*ij*_, Y*_ijk_*, Y*_ijkl_*: observed value; μ: overall mean; g_*i*_: effect of the *i*th genotype; r_*j*_: effect of the *j*th replicate (block); b*_k_* (*j*): effect of the *k*th incomplete block within the *j*th replicate; e*_k_*, e*_l_*: effect of the *k*/*l*th environment (location); b*_k_* (jl): effect of the *k*th incomplete block nested within the *j*th replication in the *l*th environment; (ge)*_ik_*, (ge)*ᵢ_l_*: genotype × environment interaction; r_*j*_(*k*), r_*j*_(*l*): replicate *j* within environment *k*/*l*; ε_*ij*_, ε*_ijk_*, ε*_ijkl_*: residual error.

In the case of CRD analysis, the classical ANOVA is generated using the *aov()* function from R's stats package (R Core Team, 2025). The model can be dynamically constructed according to the total number of factors, and it incorporates all main effects and their interactions (e.g., trait ~ factor1 × factor2 in a two‐factor experiment). The workflow also integrates post hoc mean comparisons through Tukey's honestly significant difference (HSD) test. In addition to these computations, the CRD module in PbAT includes interaction plots—an important feature that allows the user to visually examine whether factor effects are consistent.

#### Multivariate Analysis (MVA) workflow

A better understanding of the complex interrelationships among traits can exercise an effective indirect selection, and this information is critical in characterizing the broader structure of phenotypic variation (Montesinos‐López et al., 2019). The MVA workflow in PbAT was developed to guide users to achieve this objective by executing the three critical analyses, namely, PCA, correlations, and path analysis. PCA is the most versatile tool included in the MVA workflow that allows the simultaneous exploration of multiple variables. It helps in dimensionality reduction, a biplot view of factor effects, and in genetic diversity assessment. The PCA module in PbAT incorporates various functions from the FactoMineR (Le et al., 2008) and factoextra (Kassambara et al., 2026) packages. To ensure statistical robustness, the application automatically standardizes the data (scaling to unit variance) prior to computing the principal components. A correlation module was integrated into the MVA workflow to depict the relations between traits with the help of the corrplot package (Wei et al., 2017). Similarly, the path analysis function in PbAT helps to partition the correlations of a dependent factor into direct and indirect effects between a set of independent traits by incorporating necessary functions of the lavaan package (Rosseel, 2012).

#### Stability Analysis (STA) workflow

STA is one of two exclusive plant breeding–linked analytical workflows in PbAT. This workflow facilitates varietal evaluation trials to identify widely adapted or location‐specific genotypes, which is crucial for varietal development, testing, and release (Elmerich et al., 2023), based on the genotype's adaptability to the environment(s) by accounting for the GEI. Two analytical approaches, AMMI and GGE biplot, are available in PbAT for stability analysis. The AMMI approach integrates ANOVA for genotypic and environmental main effects with PCA of the GEI component (Crossa et al., 1990), whereas the GGE biplot adopts a graphical approach to visually display genotype performance coupled with GEI (Yan and Tinker, 2006). Both the AMMI and GGE biplot analyses in PbAT are implemented with the help of necessary functions in the metan package (Olivoto and Lúcio, 2020).

#### Mating Design Analysis (MDA) workflow

The MDA workflow is the second plant breeding–specific workflow in PbAT; it is designed to estimate the combining ability of genotypes for use in hybrid breeding programs. Hybrid development is among the most commercially intensive plant breeding endeavours worldwide, aimed at generating high‐yielding hybrids for large‐scale cultivation. Assessing combining ability offers essential insights for identifying superior parents, thereby strengthening the efficiency and success of hybrid development. The MDA workflow consolidates a number of commonly adopted mating designs, including all four fixed effects models of Griffing's diallel mating designs (Griffing, 1956), partial diallel, and line × tester (Table 2). The analysis quantifies the general combining ability (GCA) effects (which reflect additive gene action) and specific combining ability (SCA) effects (which reflect non‐additive gene actions and interactions). The workflow also generates ANOVA tables to partition the total variance into its genetic and environmental components, which is helpful in estimating the relative contribution of GCA and SCA error toward trait variation. The Mating Design result tabs display corresponding model equations for each of these mating designs, alongside the summary notes which detail the significance of GCA and SCA, identify the best general combiners among the parental lines of hybrids, and indicate the superior crosses with the largest SCA effects.

**Table 2:** Model equations applied in PbAT for the analysis of mating designs, including Griffing's methods 1–4, line × tester, and partial diallel approaches.

Mating design	Model equation	Data included	Reference
Griffing method 1	$Y_{\mathrm{ij}}=\;\mu +g_{i}+g_{j}+s_{\mathrm{ij}}+r_{\mathrm{ij}}+e_{\mathrm{ij}}$	Parents, F_1_s, and reciprocals	Griffing (1956)
Griffing method 2	$Y_{\mathrm{ij}}=\;\mu +g_{i}+g_{j}+s_{\mathrm{ij}}+e_{\mathrm{ij}}$	Parents and F_1_s	
Griffing method 3	$Y_{\mathrm{ij}}=\;\mu +g_{i}+g_{j}+s_{\mathrm{ij}}+r_{\mathrm{ij}}+e_{\mathrm{ij}}$	F_1_s and reciprocals	
Griffing method 4	$Y_{\mathrm{ij}}=\;\mu +g_{i}+g_{j}+s_{\mathrm{ij}}+e_{\mathrm{ij}}\;$	F_1_s only	
Line × tester	$Y_{\mathrm{ijk}}=\;\mu +g_{i}+g_{j}+s_{\mathrm{ij}}+r_{k}+e_{\mathrm{ijk}}$	Crosses between lines and testers (F_1_) (optionally parental data)	Kempthorne (1957)
Partial diallel	$Y_{\mathrm{ij}}=\;\mu +g_{i}+g_{j}+s_{\mathrm{ij}}+e_{\mathrm{ij}}$	A subset of F_1_ crosses	Kempthorne and Curnow (1961)

*Note*: μ: overall mean; g_*i*_, g_*j*_: general combining ability (GCA) effects of parent *i* and *j*; s_*ij*_: specific combining ability (SCA) effect for the cross between parent *i* and *j*; r_*ij*_: reciprocal effect (difference between cross *i*×*j* and *j*×*i*); r_*k*_: effect of the *k*th replication; e_*ij*_ and e_*ijk*_: random error associated with the observation.

### Case study: Analyzing a multi‐environment maize trial with PbAT

To demonstrate and test the practical utility of PbAT, a case study was conducted by sourcing a publicly available dataset from the Genomes to Fields (G2F) Initiative (Genomes to Fields, 2025). For this demonstration, we have selected the Yellow Stripe (YS) experiment (a subset of the 2023 dataset), which included 25 maize genotypes evaluated across eight locations for seven key agronomic traits. The trials were conducted adopting an RCBD with two replications. The purpose of this case study was to evaluate an analytical pipeline that spanned from initial data exploration to final decision support, aimed at identifying high‐yielding and stable hybrids across eight locations.

After uploading the dataset, the spatial trait explorer was used to generate heatmaps for grain yield in each environment (Figure 2A). These spatial gradients were minimal in most of the trials. Outlier analysis (based on the IQR) detected a small number of outliers across traits in the data set, and following a rigorous manual inspection (based on biological implausibility indicative of data entry/measurement errors and severe discrepancy with the second replicate of the same genotype indicating a plot‐specific error), 12 extreme data points were removed. The curated data were then loaded into the application, and descriptive statistics and boxplots were generated for the key traits. The descriptive analysis revealed a wider range of phenotypic variation in the case of most of the traits (Figure 2B).

**Figure 2:**
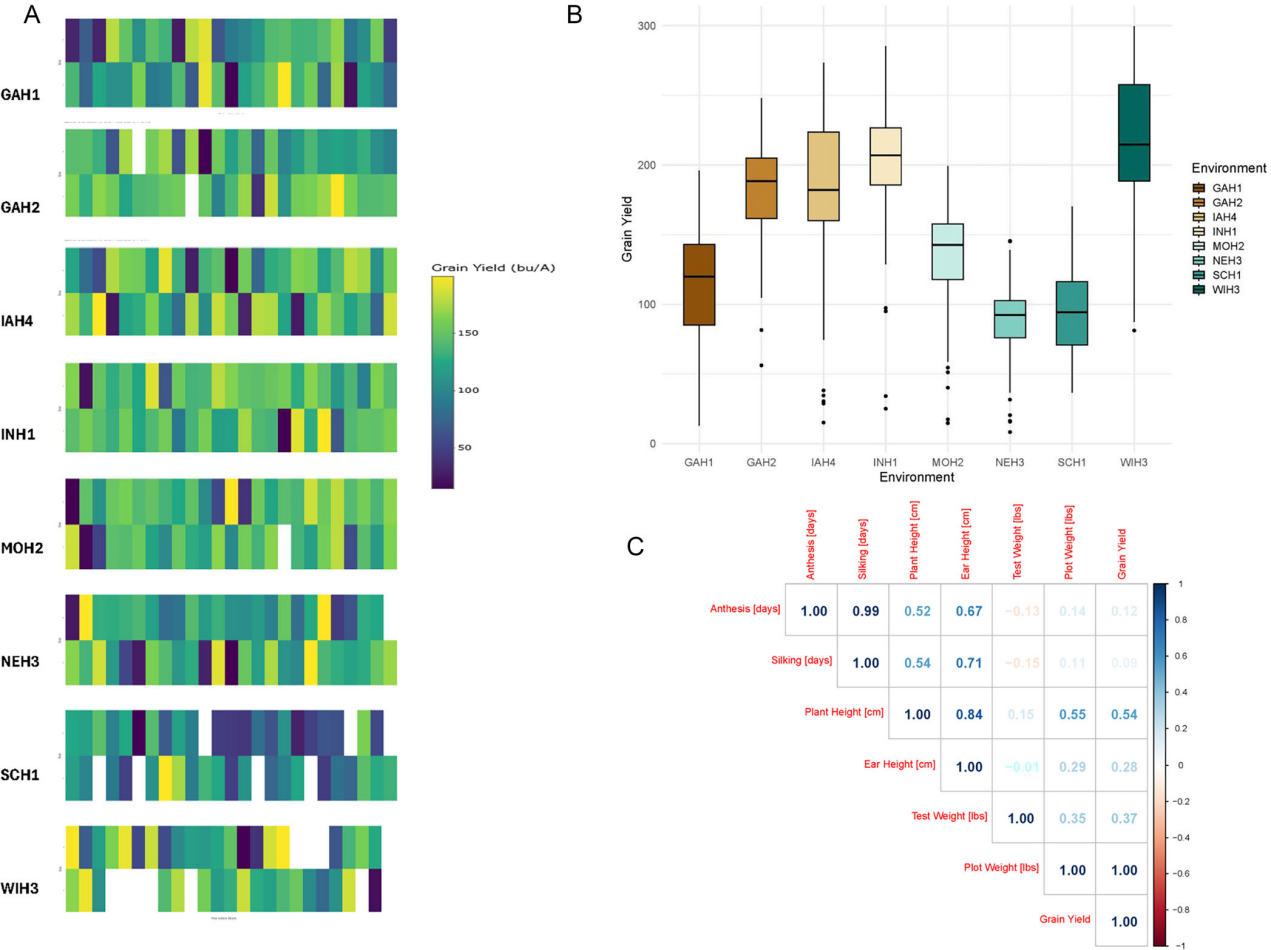
Exploratory data analysis and trait association plots generated by PbAT. (A) Spatial distribution heatmap indicating raw grain yield variability across the field layout for the identification of spatial trends. (B) Boxplots demonstrating the distribution of grain yield across environments. (C) Graphical representation of pairwise correlations between grain yield and secondary traits, where color intensity indicates the magnitude of the correlation coefficient.

In the next step, we carried out model analysis by treating the genotype as a random effect to estimate pooled and environment‐specific BLUP values. PbAT fitted the following linear mixed model for the corresponding multi‐environmental RCBD data:

(Trait~(1|Genotype)+(1|Environment)+(1|Genotype:Environment)+(1|Block:Environment))

The analysis revealed that genotype and the GEI were significant for the yield trait (*P* < 0.05), as confirmed by an LRT. This indicated significant genetic differences among hybrids and GEI across locations. The combined BLUP values across locations generated from model analysis were further used in analysis 2 as direct input from the menu and to estimate the correlation between traits. The analysis detected that a significant positive correlation exists between anthesis and plant height, plot weight, and grain yield and anthesis and silking with plant height (Figure 2C). The biplot generated by PCA has demonstrated the variations of these genotypes with respect to the investigated traits (Figure 3A).

**Figure 3:**
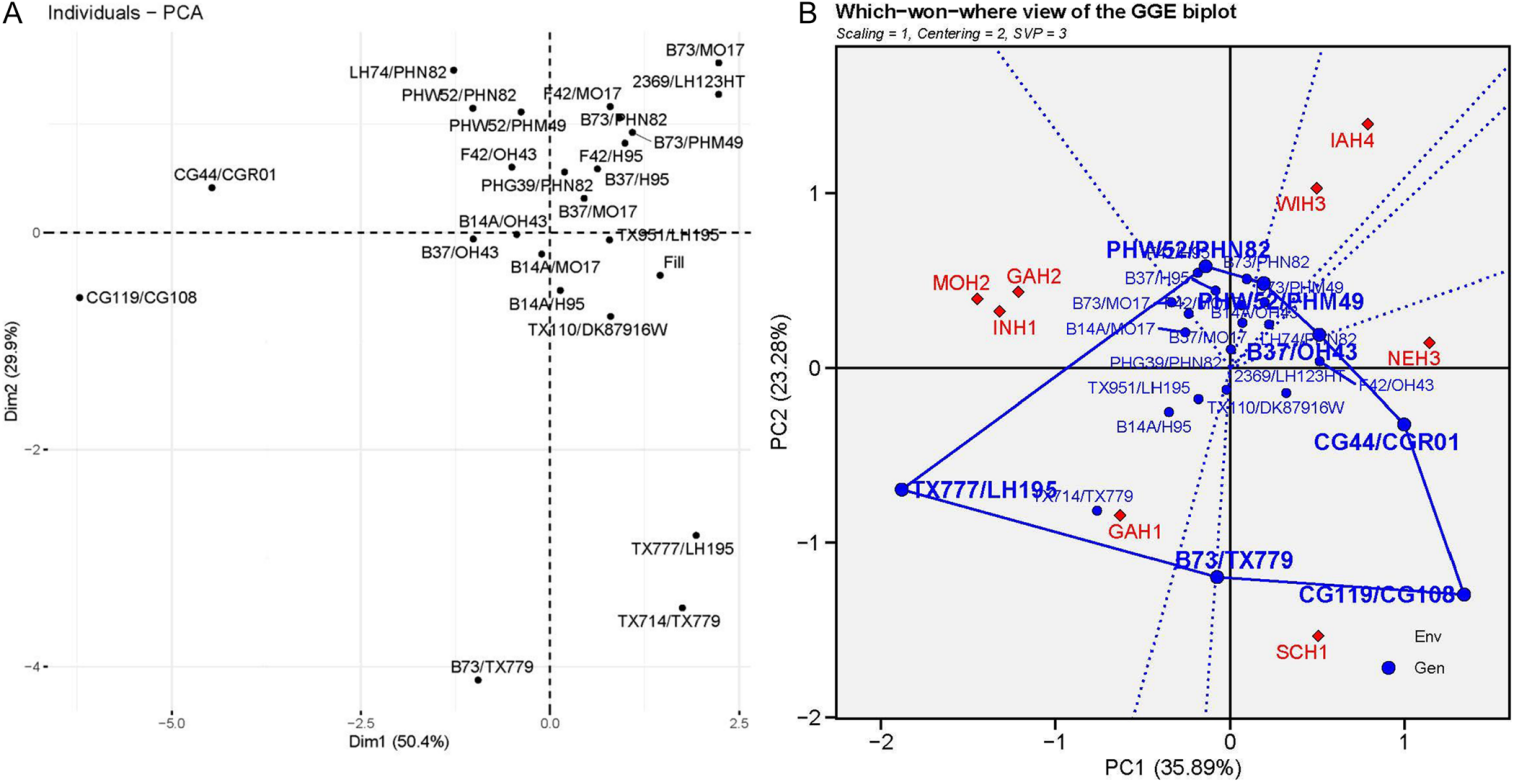
Multivariate assessment of genotypic performance and adaptability. (A) Principal component analysis (PCA) biplot depicting genotypic clustering based on multiple phenotypic traits. (B) GGE biplot (which‐won‐where plot) depicting a polygon view partitioning test environments into sectors to identify distinct mega‐environments and the specific “winning” genotypes best adapted to each sector.

The location‐specific BLUP values for grain yield were further used in the stability analysis workflow. The GGE biplot analysis generated three distinct plots, among which the “which‐won‐where” plot revealed clear differences in the superior hybrids across distinct environments. The hybrid PHW52/PHN82 performed best in the mega‐environment comprising MOH2, GAH2, and INH1. In the second mega‐environment, consisting of IAH4 and WIH3, PHW52/PHM49 was detected as the top‐performing hybrid. The hybrids TX777/LH195 and CG119/CG108 were identified as the best‐performing hybrids in the GAH1 and SCH1 environments, respectively (Figure 3B). The mean vs. stability plot revealed B73/MO17 and F42/MO17 as the most stable and high‐yielding hybrids across all test locations. The input data file is provided in Appendix S2, and the completely analyzed data are provided in Appendix S1.

This case study demonstrates the potential of PbAT in supporting breeders to seamlessly execute each analytical objective from data preprocessing, descriptive analysis, multi‐environment modeling, multivariate analysis, and genotype stability visualization. The analytical pipeline successfully identified both specific and generally adopted hybrids across the environments.

## DISCUSSION

The advancement of high‐throughput phenotyping and complex experimental designs has necessitated the use of robust statistical environments, such as R, in plant breeding. However, the steep learning curve associated with command‐line programming remains a significant hurdle for many breeders and students. While several GUI‐based tools exist, they often suffer from limited scope and lack of maintenance (Table 3). Unlike commercial software packages such as GenStat or SAS, which offer comprehensive features but are often cost‐prohibitive for researchers in developing nations, PbAT is fully open‐source and free to use.

**Table 3:** Comparative analysis of PbAT against existing commercial and open‐source plant breeding analytical tools.

Software/tool	Interface requirement	Learning curve	Scope of functionality	Maintenance and availability	Licensing model
R packages (General)	Programming/scripting required	Steep; requires coding expertise	High (Dependent on user skill)	Active; community‐supported	Open access
META‐R	GUI	Moderate	Restricted; specific workflows only	Low; updates are infrequent	Open access
AGD‐R	GUI	Moderate	Restricted; focus on genetic designs	Low; limited availability	Open access
STAR	GUI	Moderate	Restricted; limited modules	Low; support discontinued	Open access
PBTools	GUI	Moderate	Restricted; limited modules	Low; support discontinued	Open access
SAS	Syntax/programming required	Steep; complex syntax	Comprehensive	Active; professional support	Commercial (High cost)
GenStat	GUI and syntax options	Moderate	Comprehensive	Active; professional support	Commercial (High cost)
ASReml	Syntax/programming required	Steep; specialized syntax	Specialized (Mixed models)	Active; professional support	Commercial (High cost)
SPSS	GUI (Syntax optional)	Moderate	General statistics (Non‐breeding specific)	Active; professional support	Commercial (High cost)
PbAT	Code‐free and interactive GUI	User‐friendly	Comprehensive (End‐to‐end)	Active	Open access

PbAT is built on the modern Shiny framework, ensuring cross‐platform compatibility and ease of deployment via web browsers, and can also be accessed as an R package that can operate offline once installed. Unlike commercially available software, PbAT provides an open‐access alternative that democratizes access to advanced statistical methods, which are often absent in basic open‐source statistical packages. Furthermore, what distinguishes PbAT from general‐purpose statistical GUIs is that the software is specifically tailored for data analytic functions in plant breeding.

Another unique feature of PbAT that distinguishes it from purely analytical tools is its focus on pedagogical support. The application not only generates the outputs of statistical results but also provides automated, plain‐language interpretations and displays the underlying model equations. This feature addresses the black box nature of many statistical software packages, fostering a deeper understanding of quantitative genetics principles among students and early‐career researchers. By integrating visualization tools like the sTEX and outlier detection directly into the workflow, the platform encourages rigorous data curation practices before analysis begins. These functions are rarely integrated into data analysis workflows.

The current version of PbAT is built to focus exclusively on phenotypic data analysis. We acknowledge that modern breeding programs increasingly rely on genomic data to accelerate genetic gain. Therefore, we recognize the necessity of integrating high‐throughput genomic information (Sinha et al., 2021). The future development and maintenance of PbAT will focus on addressing these limitations to support Breeding 4.0 capabilities (Wallace et al., 2018). Additionally, to enhance the precision of field trials, immediate future versions of PbAT will expand its capabilities to include several other experimental designs being adopted by plant breeders, such as row–column designs and partially replicated (p‐rep) and other sparse testing designs. Users processing extremely large datasets (e.g., thousands of genotypes across dozens of environments) may experience performance latency inherent to local R/Shiny processing limitations, particularly in the web‐based version. This issue can be addressed by using the R package version of this software.

To ensure long‐term utility, PbAT is maintained as an open‐source project on GitHub (https://github.com/abhijithkpgen/PbAT). This open architecture allows the community to contribute new modules and ensures the software can evolve alongside advancements in R statistical packages. The package will follow semantic versioning, with documentation and example datasets continuously updated to support the user community.

### Conclusions

PbAT consolidates diverse statistical and graphical workflows into a unified, open‐access platform that streamlines plant breeding analytics from trial design to decision support. The application attempts to address the bottlenecks of traditional R packages and proprietary software. As an end‐to‐end pipeline, PbAT offers the functionalities of trial design, trait exploration, experimental and mating design analyses, stability analysis, and multivariate analyses. These integrated tools can help breeders and students process and transform the raw data into a chain of connected statistical analyses within a unified environment. Alongside the computations, the application also integrates automated interpretations, model equations, and publication‐ready graphical outputs. This approach not only supports data‐driven decision‐making in plant breeding but also could play a major role as a pedagogical resource for capacity building in the subject area. We have demonstrated the utility and versatility of this platform through a case study illustrating an end‐to‐end analysis of maize multi‐environment trials. The platform allows researchers to focus on biological insights and breeding decisions rather than coding or computation, thereby fostering efficiency, reproducibility, and inclusivity in plant breeding research. PbAT is available as a web application and R package, and this ensures flexibility and scope for its broad adoption as a vital tool to accelerate the development of improved crop varieties.

## AUTHOR CONTRIBUTIONS

K.P.A. conceived the project, designed the study, and led the development of the PbAT platform. K.K.V., R.K.E., R.K.T., V.M., R.K.S., and S.G.K. contributed to the methodological framework and provided domain expertise. K.P.A., K.K.V., and K.T.R. implemented the analytical workflows and conducted the case study. K.P.A. drafted the manuscript. All authors contributed to reviewing and editing the manuscript, and all authors approved the final version of the manuscript.

## Supporting information


**Appendix S1:** Supplementary tables and figures for “PbAT: A user‐friendly R/Shiny platform for data‐driven decision support in crop improvement.”

**Appendix S2:** Raw phenotypic dataset from the Genomes to Fields (G2F) 2023 multi‐environment maize trials, used as input for PbAT analyses.

## Data Availability

The PbAT source code and example datasets are openly available on GitHub at https://github.com/abhijithkpgen/PbAT. PbAT is released under an open‐source license (GPL 3.0). A citable release of version 1.0.5 is archived on Zenodo at 10.5281/zenodo.17020132 (Abhijith et al., 2025). The multi‐environment maize trial dataset used in the case study originates from the Genomes to Fields (G2F) initiative and is accessible through the CyVerse Data Commons at 10.25739/rzzy‐3n27.
