# Supplementary material for: PbAT: A user‐friendly R/Shiny platform for data‐driven decision support in crop improvement
Source: Appl Plant Sci. 2026 Jun 29;14(5):e70068. doi: 10.1002/aps3.70068 (PMC13720341; doi:10.1002/aps3.70068)
Supplement: Supplementary file 1 [file APS3-14-e70068-s002.pdf]

DOI 10.1002/aps3.70068

Appendix S1. Supplementary tables and figures for "PbAT: A user-friendly R/Shiny platform for data-driven decision support in crop improvement."

Table S1: List of all packages including supporting packages and dependencies used in PbAT v1.05

**Serial Number**	***Package Name***	**Version**	**Purpose**
1	*shiny*	1.11.1	Interactive web application framework
2	*shinyjs*	2.1.0	Performing JavaScript operations in Shiny
3	*bslib*	0.9.0	Customizing Bootstrap themes for UI
4	*waiter*	0.2.5	Loading screens and progress bars
5	*DT*	0.34.0	Interactive data tables
6	*htmlwidgets*	1.6.4	JavaScript bindings for R
7	*zip*	2.3.3	File compression and downloading
8	*dplyr*	1.1.4	Data manipulation and transformation
9	*tidyr*	1.3.1	Data tidying and reshaping
10	*readr*	2.1.5	Reading rectangular data (CSV files)
11	*tibble*	3.3.0	Modern data frame handling
12	*purrr*	1.1.0	Functional programming tools
13	*broom*	1.0.10	Converting statistical objects to tidy data
14	*rlang*	1.1.6	Core language features for tidy evaluation
15	*ggplot2*	4.0.0	Static data visualization
16	*plotly*	4.11.0	Interactive data visualization
17	*RColorBrewer*	1.1-3	Color palettes for plots
18	*colourpicker*	1.3.0	Color selection input widget
19	*corrplot*	0.95	Visualization of correlation matrices
20	*factoextra*	1.0.7	Extracting and visualizing multivariate results
21	*scales*	1.4.0	Scale functions for visualization mapping
22	*lme4*	1.1-37	Fitting linear mixed-effects models
23	*lmerTest*	3.1-3	Hypothesis testing for mixed models
24	*emmeans*	1.11.2-8	Estimated marginal means and post-hoc tests
25	*car*	3.1-3	Companion to Applied Regression (ANOVA)
26	*FactoMineR*	2.12	Multivariate exploratory data analysis (PCA)
27	*lavaan*	0.6-19	Latent variable analysis (SEM / Path Analysis)
28	*metan*	1.19.0	Multi-environment trial analysis and stability
29	*tidySEM*	0.2.9	Tidy tools for structural equation modeling
30	*future*	1.67.0	Unified parallel and distributed processing

DOI 10.1002/aps3.70068

Table S2: Summary statistics of grain yield across environments in the multi-environment maize trial dataset used for PbAT case study analysis.

Environment	Mean(g)	SD	SE	CV	Min(g)	Max(g)	N
**GAH1**	113.44	46.81	6.62	41.26	12.9	195.97	50
**GAH2**	179.71	38.62	5.57	21.49	56.21	248.12	48
**IAH4**	176.17	66.87	9.46	37.96	15.13	273.37	50
**INH1**	198.31	51.1	7.23	25.77	25.09	285.38	50
**MOH2**	132.14	41.64	5.95	31.51	14.73	199.22	49
**NEH3**	87.18	33.79	4.83	38.76	8.3	145.55	49
**SCH1**	95.18	30	4.57	31.52	36.55	170.29	43
**WIH3**	214.71	59	9.45	27.48	81.2	299.53	39

Values represent the mean yield (Mean), standard deviation (SD), standard error of the mean (SE), coefficient of variation (CV, %), minimum observed value (Min), maximum observed value (Max), and the number of observations (N) for each environment.

Table S3. Model comparison for grain yield in the multi-environment maize trial dataset.

Source	npar	logLik	AIC	LRT	Df	Pr(>Chisq)
Baseline Model (for comparison)	6	-1941.86	3895.725	NA	NA	NA
(1 | Pedigree)	5	-1969.7	3949.391	55.666	1	0
(1 | Field.Location)	5	-1953.13	3916.26	22.534	1	0
(1 | Pedigree:Field.Location)	5	-1951.81	3913.612	19.887	1	0
(1 | Replicate:Field.Location)	5	-1942.2	3894.398	0.673	1	0.412

The table reports log-likelihood (logLik), Akaike Information Criterion (AIC), and likelihood ratio test (LRT) statistics with associated degrees of freedom (Df) and significance values (Pr(>Chisq)) for evaluating alternative models relative to the baseline.

DOI 10.1002/aps3.70068

Table S4. Variance component estimates for grain yield across environments in the multi-environment maize trial dataset.

Source	Variance	Percentage
Pedigree:Field.Location	459.588	10.20%
Pedigree	726.586	16.10%
Replicate:Field.Location	21.886	0.50%
Field.Location	2278.098	50.50%
Residual	1025.2	22.70%

Table S5. Best linear unbiased predictors (BLUPs) of grain yield for maize genotypes across environments in the Genomes to Fields multi-environment trial dataset.

Pedigree	BLUP_Combined	BLUP_GAH1	BLUP_GAH2	BLUP_IAH4	BLUP_INH1	BLUP_MOH2	BLUP_NEH3	BLUP_SCH1	BLUP_WIH3
2369/LH123HT	182.155	170.554	140.966	159.937	148.184	156.478	143.775	150.722	156.006
B14A/H95	141.353	153.448	152.977	143.85	159.664	155.954	150.599	152.41	131.911
B14A/MO17	148.211	132.562	157.197	159.207	154.703	167.245	164.268	147.366	122.603
B14A/OH43	151.088	155.139	148.464	167.439	147.975	153.409	156.298	132.582	145.665
B37/H95	154.73	132.105	151.979	147.764	160.978	154.541	150.177	145.757	165.974
B37/MO17	157.071	142.259	150.421	157.455	163.053	145.54	155.972	144.971	151.084
B37/OH43	136.758	127.292	150.37	148.947	148.511	142.116	158.269	156.824	165.577
B73/MO17	194.266	160.863	156.584	167.512	158.618	147.587	128.628	144.404	170.086
B73/PHM49	175.538	154.111	141.135	170.988	160.369	150.538	162.853	134.87	147.574
B73/PHN82	173.489	153.167	146.421	153.866	149.4	158.071	162.052	131.303	166.86
B73/TX779	107.707	154.851	157.931	100.367	133.83	151.274	145.92	184.505	NA
CG119/CG108	90.277	164.158	127.34	143.967	130.474	123.319	169.082	179.429	130.738
CG44/CGR01	115.678	142.83	141.602	156.333	136.106	130.517	162.061	163.648	151.476
F42/H95	163.883	141.157	156.363	168.9	157.918	152.979	145.013	139.767	152.969
F42/MO17	176.772	154.163	160.376	167.163	145.075	152.337	131.704	148.611	163.788
F42/OH43	159.051	129.902	156.403	164.783	137.364	143.95	149.79	170.257	159.558
LH74/PHN82	165.274	162.823	154.527	155.291	137.139	145.626	153.326	138.475	168.738
PHG39/PHN82	161.409	161.426	155.504	160.892	142.361	149.632	148.976	143.855	NA

DOI 10.1002/aps3.70068

PHW52/PHM49	165.439	140.997	150.146	156.764	149.935	149.762	149.936	143.869	174.64
PHW52/PHN82	164.469	137.858	162.444	158.407	150.315	157.185	158.062	137.451	153.712
TX110/DK87916W	143.171	152.718	139.301	162.735	155.149	146.619	159.996	150.305	135.141
TX714/TX779	118.008	158.602	154.569	99.092	157.035	163.089	136.027	166.781	NA
TX777/LH195	137.908	180.462	166.979	103.549	187.841	169.002	131.598	144.093	115.11
TX951/LH195	136.761	149.946	146.16	NA	158.313	150.493	133.915	157.377	NA

Missing values (NA) indicate cases where predictions could not be obtained for a given environment.

Figure S1. GGE biplots of maize multi-environment trial data. (A) Discriminativeness vs. representativeness of test environments. (B) Mean performance vs. stability of genotypes across environments.

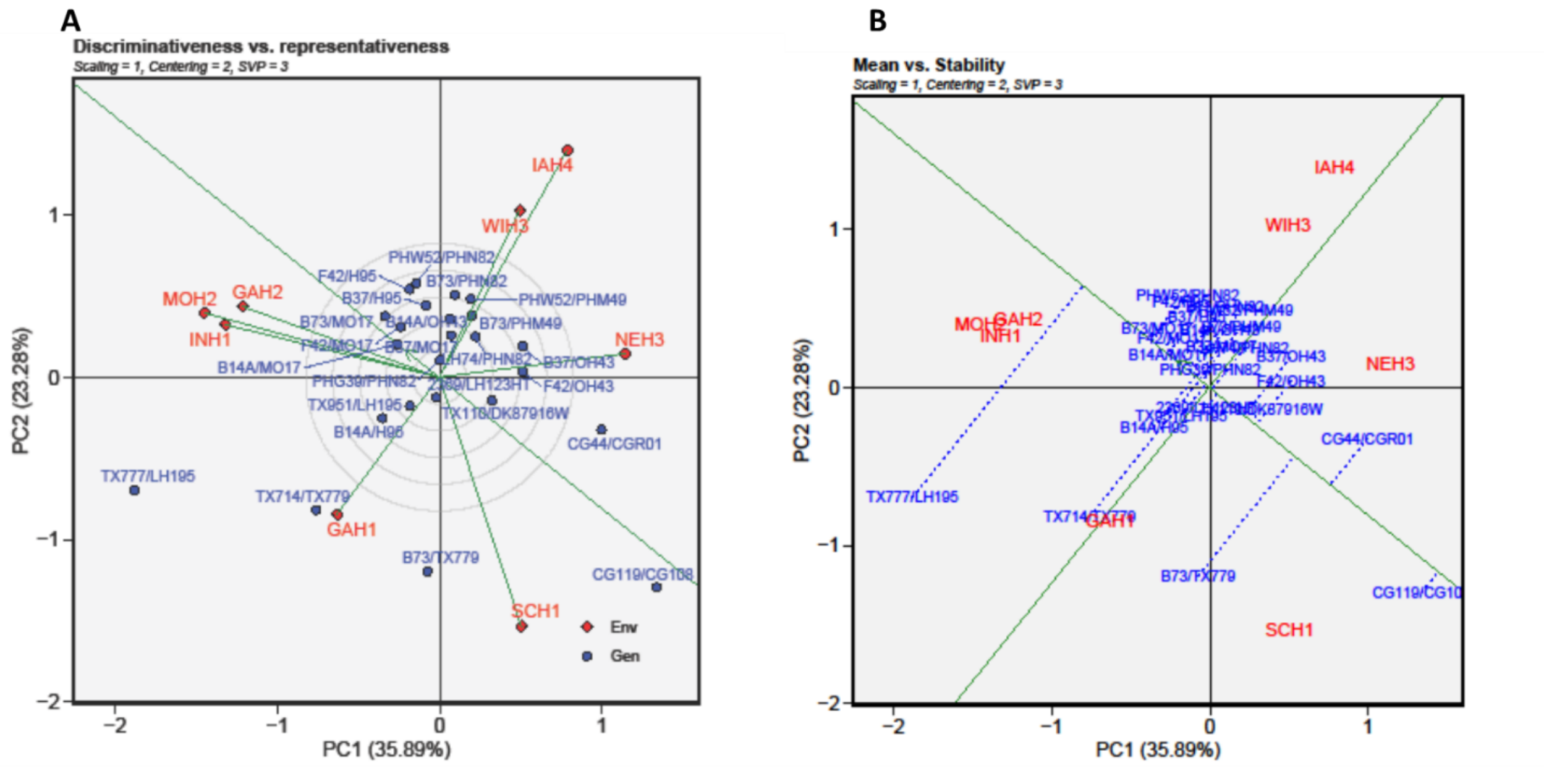

PC1 and PC2 represent the first two principal components explaining the majority of the genotype × environment interaction variance. Environments are shown in red and genotypes in blue.

DOI 10.1002/aps3.70068

Figure S2. QQ plots of residuals for grain yield across eight test environments.

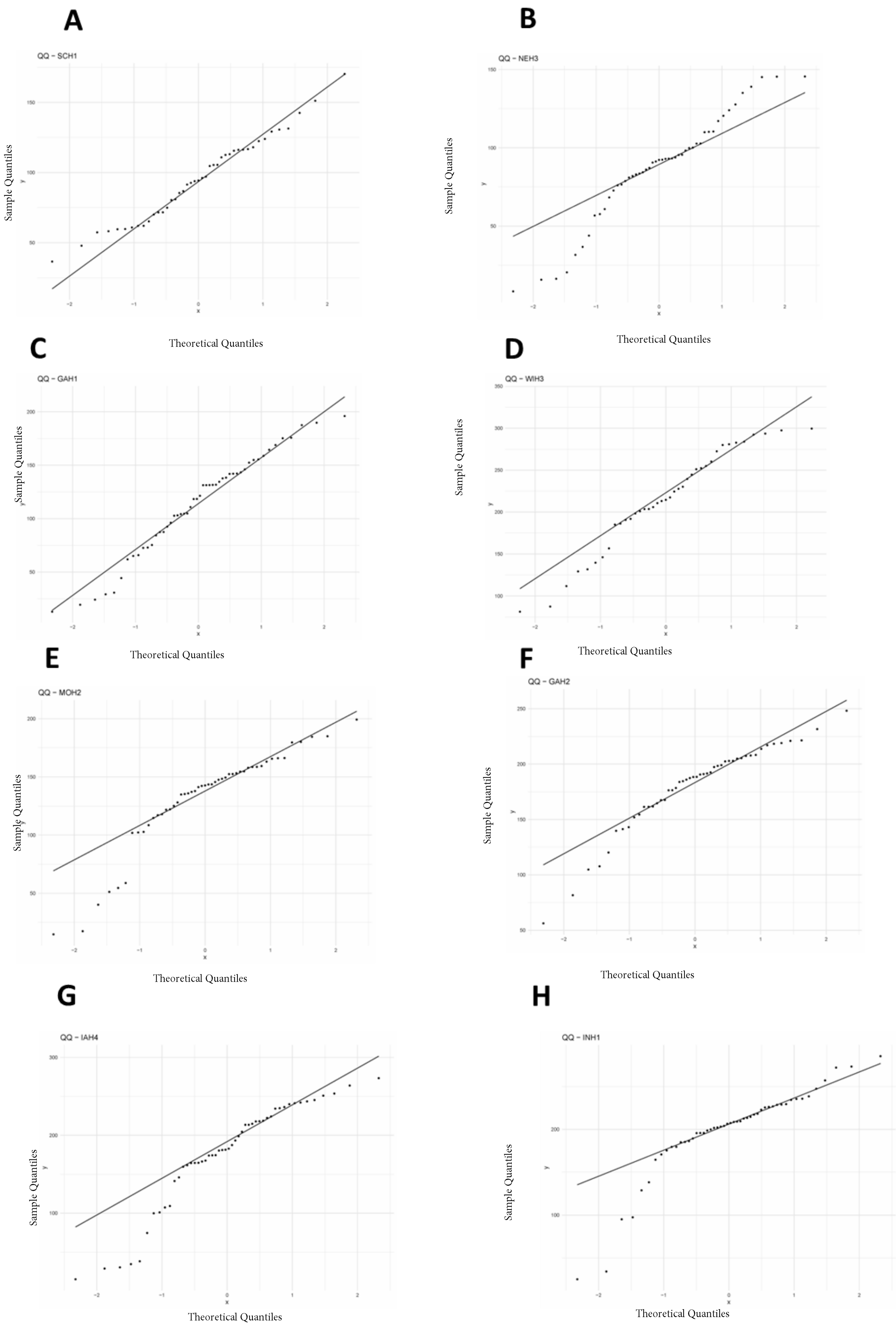

(A) SCH1, (B) NEH3, (C) GAH1, (D) WIH3, (E) MOH2, (F) GAH2, (G) IAH4, and (H) INH1.
